# Curcumin and N-Acetylcysteine Nanocarriers Alone or Combined with Deferoxamine Target the Mitochondria and Protect against Neurotoxicity and Oxidative Stress in a Co-Culture Model of Parkinson’s Disease

**DOI:** 10.3390/antiox12010130

**Published:** 2023-01-05

**Authors:** Leah Mursaleen, Stefanie Ho Yi Chan, Brendon Noble, Satyanarayana Somavarapu, Mohammed Gulrez Zariwala

**Affiliations:** 1 Centre for Nutraceuticals, School of Life Sciences, University of Westminster, 115 New Cavendish Street, London W1W 6UW, UK; 2 Department of Pharmaceutics, UCL School of Pharmacy, 29-39 Brunswick Square, London WC1N 1AX, UK; 3 Cure Parkinson’s Trust, 120 New Cavendish Street, Fitzrovia, London W1W 6XX, UK

**Keywords:** curcumin, n-acetylcysteine, deferoxamine, oxidative stress, Parkinson’s disease, neurodegeneration, iron, blood-brain barrier, Transwell^®^ model, hCMEC/D3, SH-SY5Y cells

## Abstract

As the blood-brain barrier (BBB) prevents most compounds from entering the brain, nanocarrier delivery systems are frequently being explored to potentially enhance the passage of drugs due to their nanometer sizes and functional characteristics. This study aims to investigate whether Pluronic^®^ F68 (P68) and dequalinium (DQA) nanocarriers can improve the ability of curcumin, n-acetylcysteine (NAC) and/or deferoxamine (DFO), to access the brain, specifically target mitochondria and protect against rotenone by evaluating their effects in a combined Transwell^®^ hCMEC/D3 BBB and SH-SY5Y based cellular Parkinson’s disease (PD) model. P68 + DQA nanoformulations enhanced the mean passage across the BBB model of curcumin, NAC and DFO by 49%, 28% and 49%, respectively (*p* < 0.01, n = 6). Live cell mitochondrial staining analysis showed consistent co-location of the nanocarriers within the mitochondria. P68 + DQA nanocarriers also increased the ability of curcumin and NAC, alone or combined with DFO, to protect against rotenone induced cytotoxicity and oxidative stress by up to 19% and 14% (*p* < 0.01, n = 6), as measured by the MTT and mitochondrial hydroxyl radical assays respectively. These results indicate that the P68 + DQA nanocarriers were successful at enhancing the protective effects of curcumin, NAC and/or DFO by increasing the brain penetrance and targeted delivery of the associated bioactives to the mitochondria in this model. This study thus emphasises the potential effectiveness of this nanocarrier strategy in fully utilising the therapeutic benefit of these antioxidants and lays the foundation for further studies in more advanced models of PD.

## 1. Introduction

Parkinson’s disease (PD) is one of the most common progressive neurodegenerative diseases [1,2,3,4]. It is characterised by a marked and continued loss of dopaminergic neurons in the substantia nigra of the brain [1,2]. Although the aetiology of PD is not yet fully understood, several studies have suggested that excessive free iron and the related oxidative stress within these neurons play a major role in both the development and progression of the disease [5,6,7,8,9]. In PD, the ability of free iron to react with hydrogen peroxide via the Fenton reaction, coupled with the reduced levels of endogenous protective antioxidants, such as glutathione, drives the continual production of toxic hydroxyl radicals [10,11,12,13,14,15]. Such increased levels of hydroxyl radicals within mitochondria results in sustained oxidative damage to proteins, lipids and DNA and eventual cell death [16,17]. 

Compounds such as curcumin and n-acetylcysteine (NAC) have generated interest as potential disease-modifying treatments for PD due to their antioxidant capabilities [4,18,19,20,21,22,23]. Equally, the potential of iron chelators such as deferoxamine (DFO) have also been investigated as a preventative strategy to reduce the production of hydroxyl radicals [24,25,26]. Numerous *in vitro* and *in vivo* studies have indicated neuroprotective effects of DFO, curcumin and NAC individually [18,19,20,21,22,23,24,25,26], and it is hypothesised that the combination of iron chelators and antioxidants may exert a more potent effect due to the two-pronged approach to combat oxidative stress by limiting the availability of detrimental free iron whilst neutralising any free radicals that may be generated [27]. However, the therapeutic values of such compounds are reduced by issues such as low bioavailability, minimal brain penetrance, and lack of organelle targeting [13,28,29,30]. 

Nanocarriers have demonstrated potential as targeted delivery systems to counter issues of bioavailability and to enhance delivery across biological membranes, including the blood brain barrier (BBB) [31,32]. Polymeric micelles are considered particularly advantageous for brain delivery due to characteristics such as small particle size (10–200 nm), high water-solubility, high association efficiency, as well as relatively low toxicity [33,34,35,36,37]. The amphiphilic polymer Pluronic^®^ F68 (P68) combined with the mitochondria targeting agent, dequalinium (DQA), have been successfully used to develop micellar nanocarriers of curcumin and NAC, alone or in combination with DFO [38,39]. Previous studies have shown that free and P68 + DQA nanoformulated preparations of curcumin, NAC and/or DFO, are protective against rotenone induced oxidative stress and cytotoxicity in the neuronal SH-SY5Y cells [38,39]. Nanoformulations containing the mitochondrial targeting agent DQA also demonstrated increased ability to protect against the rise in mitochondrial hydroxyl radicals following rotenone treatment, evidencing indirectly their potential to target the mitochondria [32,40,41]. The ability to target the mitochondria is particularly desirable, since mitochondria are the main site of oxidative stress [12,14,15,42]. 

However, as SH-SY5Y cells were directly exposed to the treatments in these studies, it is not established whether the effects would be replicated *in vivo*, where the ability of drugs to treat neurological conditions are limited due to the primary challenge of permeating across the BBB. The BBB is known to prevent the passage and activity of 98% of potential neuropharmaceuticals [43] and although curcumin, DFO and NAC may have some brain penetrance, they are unlikely to access the brain in sufficiently high concentrations to have a significant effect on PD progression. In addition, the ability of these nanoformulations to directly and specifically target the mitochondria has also not been determined previously in this context.

Therefore, this study aims to investigate whether P68 + DQA nanoformulations improve the ability of curcumin and NAC, alone and in combination with DFO, to access the brain and target mitochondria in a biomimetic manner by evaluating the effects of each drug in a combined Transwell^®^ hCMEC/D3 BBB and SH-SY5Y cellular model. It will also evaluate whether the protective effects of the free and formulated drugs against rotenone induced cytotoxicity and oxidative stress are retained following passage across the BBB model. A significant advantage of using such a co-culture *in-vitro* model is that it allows quantitative estimation of the relative bioaccesibility of the nanoformulations and qualitative real time evaluation of their mitochondrial targeting ability thus laying the foundation for more specific future *in vivo* studies utilising the most optimum nanoformulated preparation(s).

## 2. Materials and Methods

### 2.1. Materials

Unless otherwise stated, chemicals were either analytical grade or cell culture grade. SH-SY5Y cells were from the American Type Culture Collection (ATCC CRL-2266, Manassas, VA, USA) and hCMEC/D3 cells were kindly gifted from Dr Simon McArthur (Queen Mary University of London, UK). The hCMEC/D3 cells originated from VHBio Ltd. (Gateshead, UK) (Hoyles et al., 2018). EGM-2 MV Microvascular Endothelial Cell Growth Medium-2 BulletKitTM, Hank’s Buffered Saline Solution (HBSS), HEPES Buffer (1 M), Dulbecco’s phosphate buffered saline (DPBS), fibronectin (bovine plasma) and collagen from calf skin, were supplied by Lonza (Slough, UK). Fisher Scientific (Loughborough, UK) supplied methanol (HPLC grade), L-glutamine, foetal bovine serum (FBS), Dulbecco’s Modified Eagle Medium (DMEM) Glutamax^®^, Minimum Essential Media (MEM), 100× antibiotic-antimycotic and poloxomer 68 (Pluronic^®^ F68), as well as the MitoTracker Red CMXRos kit and NucBlue™ Live ReadyProbes™ reagent. Dimethyl sulfoxide (DMSO), Thiazolyl Blue Tetrazolium Blue (MTT), Dequalinium chloride hydrate (95%), Rotenone (≥95%), Curcumin (≥94% curcuminoid content, ≥80% curcumin), Deferoxamine mesylate salt (92.5%) and N-Acetyl-L-cysteine (99%) were obtained from Sigma-Aldrich (Poole, UK). Abcam (Cambridge, UK) was the supplier of the mitochondrial hydroxyl radical detection assay kit (cat no. ab219931). Millipore (Dublin, Ireland) provided the Millex-MP sterile filters and flasks were from Nunc (Roskilde, Denmark). Corning (Fisher Scientific, Loughborough, UK) supplied the plasticware such as culture plates, pipettes, eppendorf tubes and stripettes. Purified water with a resistivity of 18.2 MΩ (Milli-Q water) was used to prepare experimental reagents.

### 2.2. Preparation of Antioxidant and/or Iron Chelator Nanoformulations

Nanoformulations were prepared using a modified thin-film hydration method [32,40]. Different ratios of P68 and DQA were dissolved in 10 mL of methanol along with the antioxidant (curcumin or NAC) and/or iron chelator (DFO) of interest (Table 1). Evaporation of methanol and production of the thin-film was achieved under vacuum using a rotary evaporator (Hei-VAP Advantage Rotary Evaporator, Heidolph, Germany) at 200 rpm and 80 °C. Distilled water (10 mL) was mixed in at 80 °C for 1–2 min and sonicated for a further 1 min using a VWR Ultrasonic cleaner bath USC300T (VWR International Limited, Lutterworth, UK) to hydrate and disperse the film. The solution was then sterile filtered (0.22 μm) to remove any unloaded antioxidant and/or iron chelator.

### 2.3. Nanoformulation Size and Surface Charge

Nanoformulation particle size distribution was measured as Z_Ave_ hydrodynamic diameter and polydispersity index. Surface charge was measured as zeta potential. These characteristics were assessed using the Zetasizer Nano ZS (Malvern Instruments, Malvern, UK).

### 2.4. Nanoformulation Drug Loading and Association Efficiency

Nanoformulation drug loading and association efficiency were studied using UV-Visible (UV-Vis) spectroscopy and calculated from the calibration curves of the free drugs, as previously described [38,39,44]. In order to achieve a theoretical concentration of each drug (10 μg/mL curcumin, 20 μg/mL DFO and 1 mg/mL NAC), the nanocarrier was dissolved in methanol and water (1:1 ratio) to release the drug(s). Curcumin, DFO and NAC content were calculated using UV-Vis spectroscopy at 423 nm, 204 nm and 234 nm, respectively. Calculation of drug loading and association efficiency were carried out using the following equations:

Drug loading (%) = (determined mass of drug within nanocarriers/mass of drug-loaded nanocarriers) × 100

Association efficiency (%) = (determined mass of drug within nanocarriers/theoretical mass of drug within nanocarriers) × 100

### 2.5. hCMEC/D3 Cell Culture

The hCMEC/D3 cerebral microvascular endothelial cell line was used to create an *in vitro* model of the BBB as previously described [44]. Briefly, hCMEC/D3 cells were grown in Microvascular Endothelial Cell Growth Medium-2 BulletKitTM (EGM-2 MV) that contains 0.1% *w/v* ascorbic acid, 5% *v/v* foetal bovine serum (FBS), 0.1% *v/v* gentamicin sulfate-amphotericin, 0.4% *v/v* human basic fibroblast growth factor, 0.1% *v/v* human recombinant insulin-like growth factor, 0.04% *v/v* hydrocortisone and 0.1% *v/v* vascular endothelial growth factor, in 5% CO_2_ and 37 °C environment until ~70% confluent. Following trypsinisation, the cells were seeded at 300,000 cells/cm^2^ into polycarbonate membrane inserts (3.0 μm pore size) of 6 and 96-well Costar Transwell^®^ plates according to the experiment being conducted. Inserts were precoated with 1:20 type 1 collagen from calf skin: DPBS for 1 h followed by 1:100 fibronectin from bovine plasma: DPBS for a further 1 h. 72 h before testing, hCMEC/D3 cells were incubated in VEGF-free media to aid promote tight junction formation [45,46,47,48].

### 2.6. Trans-Endothelial Electrical Resistance Assessment

Trans-endothelial electrical resistance (TEER) was measured to assess the resistance of the BBB model as previously described [44,49]. An epithelial Volt-Ohm meter and sterile Chopstick Electrodes were used to obtain the TEER values which are calculated from the resistance of the tissue (Ω) × membrane area (cm^2^) and therefore expressed as Ω.cm^2^. High TEER values are desired as the presence of tight junctions increases the resistance [50]. In the presence of hydrocortisone, hCMEC/D3 TEER values have been shown to reach ~300 Ω.cm^2^ [44,48,51,52]. Once seeded into Transwell^®^ plates, TEER values were therefore measured each day until a resistance in the region of 300 Ω.cm^2^ was reached before carrying out any of the BBB model passage experiments. 

### 2.7. BBB Membrane Permeability–Lucifer Yellow Assay

The integrity of the BBB model was also assessed using the Lucifer yellow permeability assay (as previously described by [52,53,54]). The lithium salt Lucifer yellow CH is a small (MW 457 Da) hydrophilic dye that is retained by the BBB and therefore presence of Lucifer yellow passing through the BBB model indicates a weak, leaky model [52]. Once a TEER value of 300 Ω.cm^2^ was reached, hCMEC/D3 cells were incubated at 37 °C for 1 h with 1.5 mL 0.1 mg/mL Lucifer yellow in HBSS + 10 mM HEPES in the apical upper compartment and 2.5 mL of HBBS in the lower basolateral chamber. Following this, the medium in the basolateral compartment and samples from the treatments added to the apical compartment were aliquoted in duplicate into a black 96 well plate in order to carry out fluorometric analysis using the Fluostar Optima Fluorescence Plate Reader (BMG LABTECH, Aylesbury, UK) at excitation 485 nm, emission at 535 nm. The results were expressed in terms of permeability in 10^–3^ cm/min with the aim of achieving a permeability between 1.2 and 0.6 × 10^–3^ cm/min as previously reported [55,56,57], as it is above this point when the barrier is considered permeable or open. The permeability coefficient (Pc) was calculated from the following equation: Pc (cm/min) = (Vb × Cb)/(Ca × A × T) where Vb is the volume in the basolateral side (μL), Cb is the final basolateral concentration of Lucifer yellow (μM), Ca is the initial apical concentration of lucifer yellow (μM), A is the membrane growth area (cm^2^), and T is the time of transport (min) [52,53].

### 2.8. Assessment of Nanocarrier BBB Permeability 

A transport assay was carried out to assess the flux of nanoformulations across the model BBB as previously described [44,58,59,60]. Each chamber was washed three times with phenol red-free HBSS carefully to avoid disturbing the hCMEC/D3 monolayer. The apical and basolateral chambers were then incubated at 37 °C for 10 min in 1 mL and 2.5 mL HBSS, respectively. Following aspiration, the apical chamber was treated for 1 h at 37 °C with 1.5 mL nanoformulated or corresponding free curcumin, NAC and/or DFO treatments (in HBSS) at a range of concentrations. The basolateral chambers were then sampled and curcumin, DFO and NAC content was calculated using UV-Vis spectroscopy (as described above) at 423 nm, 204 nm and 234 nm, respectively. TEER measurements were taken immediately after each transport assay to assess the stability of the BBB model and potential toxicity of each treatment.

### 2.9. hCMEC/D3 and SH-SY5Y Co-Culture 

As described above, the hCMEC/D3 cells were grown in T75 flasks until sufficiently confluent (~70%). The adherent cells were then seeded into the 3.0 μm pore polycarbonate membrane inserts of 6-well or 96-well Costar Transwell^®^ plates precoated with 1:20 type 1 collagen from calf skin: DPBS (1 h) and 1:100 fibronectin from bovine plasma: DPBS (1 h), at a seeding density of 300,000 cells/cm^2^. SH-SY5Y cells were grown in parallel. Once 70% confluent, adherent SH-SY5Y cells were detached from the surface of the T75 flasks via trypsinisation and seeded at 1,000,000 cells/cm^2^ into 6-well or 96-well plates. The Transwell^®^ inserts containing hCMEC/D3 cells at a membrane potential of 300 Ω.cm^2^ were placed into the relevant culture plates once the SH-SY5Y cells were confluent. The treatments and assays outlined below were then immediately carried out using this co-culture system.

### 2.10. Assessment of the Ability of Nanocarriers to Permeate the BBB and Target Mitochondria 

The ability of the curcumin nanoformulations to target mitochondria was assessed as previously described by Zupančič et al. [32]. The basolateral chamber of the 6-well co-culture system containing confluent SH-SY5Y cells was treated with 2.5 mL MEM. The apical chamber containing hCMEC/D3 cells was treated with 1.5 mL nanoformulated or corresponding free curcumin treatments (in HBSS) at 10 μM concentration and incubated for 1 h at 37 °C in a humidified incubator containing 5% CO_2_. The basolateral inserts were then removed and the SH-SY5Y cells were incubated for a further 2 h at 37 °C to allow the treatments to be internalised. Following the incubation, the treatment media was aspirated, and cells were washed with DPBS and incubated for 30 min with 100 nM solution of red fluorescent mitochondrial dye Invitrogen™ MitoTracker™ Red CMXRos in MEM. The cells were then washed with DPBS and cell nuclei were stained with NucBlue™ Live ReadyProbes™ reagent diluted in MEM. The samples were examined under a fluorescent microscope (EVOS™ FL Auto 2–Invitrogen, Fisher Scientific, Loughborough, UK) to assess mitochondrial targeting properties of curcumin-loaded nanocarriers. These experiments were carried out using curcumin and curcumin-loaded nanocarriers only due to the ability of curcumin to auto-fluoresce and be identified using a fluorescent microscope. The nuclei, mitochondria and curcumin were visualised using the DAPI (excitation: 357/44 nm, emission: 447/60 nm), CY5 (excitation: 628/40 nm, emission: 685/40 nm) and GFP (excitation: 470/22 nm, emission: 525/50 nm) objectives respectively. 

### 2.11. Assessment of the Ability of Nanocarriers to Pass the BBB and Protect against Rotenone 

The basolateral chamber containing confluent SH-SY5Y cells was treated with MEM (200 μL). 150 μL of nanoformulated or corresponding free curcumin, NAC and/or DFO treatments (in HBSS) was added to the apical chamber containing hCMEC/D3 cells at a range of concentrations and incubated for 1 h at 37 °C. Following removal of the inserts, SH-SY5Y cells were incubated at 37 °C for a further 2 h. The cells then underwent a 24 h 100 μM rotenone treatment. To assess the protective effects of the treatments against rotenone induced cytotoxicity and oxidative stress following passage across the BBB model, the MTT and mitochondrial hydroxyl radical assays were carried out, as previously described [44]. Rotenone only and MEM only treatments, without any pre-treatments, were used as controls.

The protective properties of drug-loaded nanocarriers against rotenone-induced reduction in cell viability was assessed using the MTT Assay. 20 μL of 5 mg/mL solution of MTT DPBS solution was added to the cells at 37 °C for 4 h. The resulting formazan crystals were dissolved in 100 μL of DMSO, following aspiration. The MaxQ 4000 benchtop orbital shaker (Thermo Fisher Scientific, Loughborough, UK) was used at 75 rpm for 15 min to ensure the DMSO was mixed well. A spectrophotometer (VersaMax, Molecular Devices, San Jose, CA, USA) was then used to read the absorbance at 570 nm. 

The mitochondrial hydroxyl radical detection assay was carried out in the co-culture model according to manufacturer’s protocol (ab219931; Abcam, Cambridge, UK). After the nanoformulation treatments and once the hCMEC/D3 inserts had been removed (described above), DPBS was used to wash the SH-SY5Y cells, and they were then treated at 37 °C for 1 h with 100 µL of the hydroxyl radical specific OH580 probe. The cells were then incubated with 100 µM rotenone for 24 h at 37 °C. Once washed, the cells were read on the Fluostar Optima Fluorescence Plate Reader (BMG LABTECH, Aylesbury, UK) at 540/590 nm excitation/emission.

### 2.12. Statistical Analysis

The data is provided as mean ± standard deviation (S.D.), for all experiments the mean of six replicates was calculated for each treatment. Statistical analysis of the TEER and BBB model passage data was carried out using two-way analysis of variance (ANOVA) with the Šidák multiple comparisons post hoc test. One-way ANOVA with the Dunnett’s T3 post hoc test was used for the results of each MTT and mitochondrial hydroxyl radical assay (PRISM software package, Version 8, Graphpad Software Inc., San Diego, CA, USA).

## 3. Results

All drug loaded nanoformulations exhibited high association efficiency (82–98%) with NAC formulations exhibiting 6% and 16% higher mean association efficiencies than curcumin formulations in the antioxidant only and combined antioxidant and DFO formulations (respectively), however there was no significant difference (Table 1). All drug loaded nanocarrier formulations exhibited a significantly higher mean particle size compared to the unloaded blank nanoformulation (*p* < 0.0001) (Table 1). The addition of DFO into the formulation, appeared to increase the mean association efficiency of NAC (5%) but not curcumin (−4%) (Table 1). The mean size of both the NAC (126 nm) and NAC + DFO (130 nm) P68 + DQA nanocarriers were smaller than the curcumin (183 nm) and curcumin + DFO (192 nm) nanocarriers but the DFO only nanocarrier exhibited the smallest particle size of 50 nm (Table 1). The addition of DFO to the NAC and curcumin P68 + DQA nanoformulations did not significantly alter particle size (Table 1). All nanoformulations had low polydispersity as represented by mean polydispersity indices < 0.25, which indicated that the majority of the nanocarriers within each formulation sample were of similar size (Table 1). The mean surface charge of all drug loaded nanocarriers were moderately positive (4–9 mV) but each drug loaded nanoformulation had a higher surface charge compared to the blank unloaded nanoformulation, which exhibited a slightly negative charge (−0.78 mV) (Table 1).

**Figure 1:**
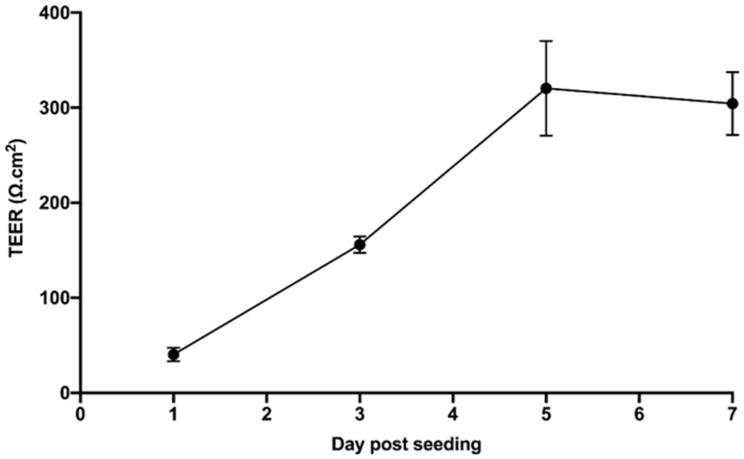
Mean TEER of hCMEC/D3 cell monolayers grown on 3.0 µm Transwell^®^ inserts on days 1, 3, 5 and 7 post cell seeding.

The mean TEER of hCMEC/D3 cell monolayers grown on Transwell^®^ inserts was shown to peak at 320 Ω.cm^2^ on day five post seeding, falling to 304 Ω.cm^2^ by day seven (Figure 1). On day five post seeding, the permeability of the hCMEC/D3 monolayers were consistently below 1.2 × 10^−3^ cm/min (0.91 ± 0.13 × 10^−3^ cm/min).

**Figure 2:**
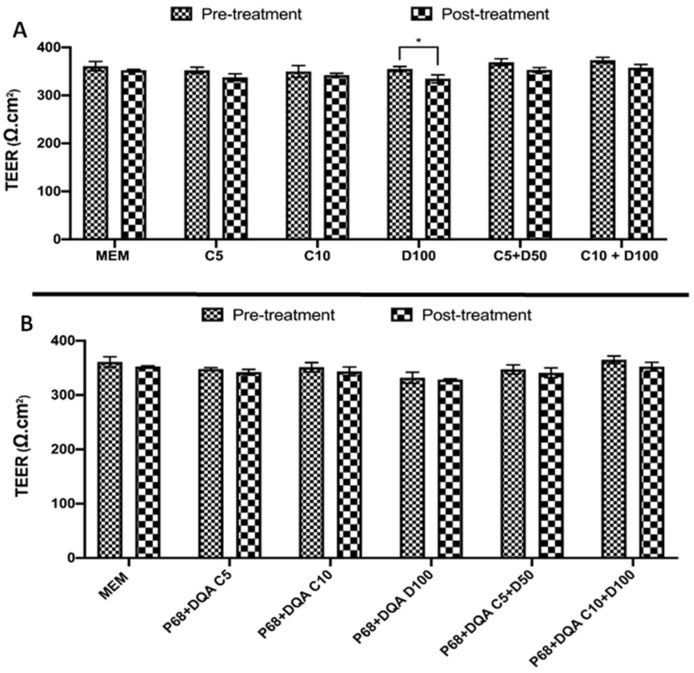
(**A**) Mean TEER of hCMEC/D3 cell monolayers on day 3 post seeding before (pre-treatment) and after (post-treatment) treatment with free 5 or 10 µM curcumin (C5 and C10), 100 µM DFO (D100) or the combination of 5 µM or 10 µM curcumin with 50 µM or 100 µM DFO (C5 + D50 and C10 + D100, respectively). (**B**) Corresponding TEER results pre- and post-treatment with P68 + DQA nanoformulations of curcumin, DFO and combined curcumin and DFO. * represents significance values when comparing the pre- and post-treatment TEER values for a given treatment condition (* *p* < 0.05).

**Figure 3:**
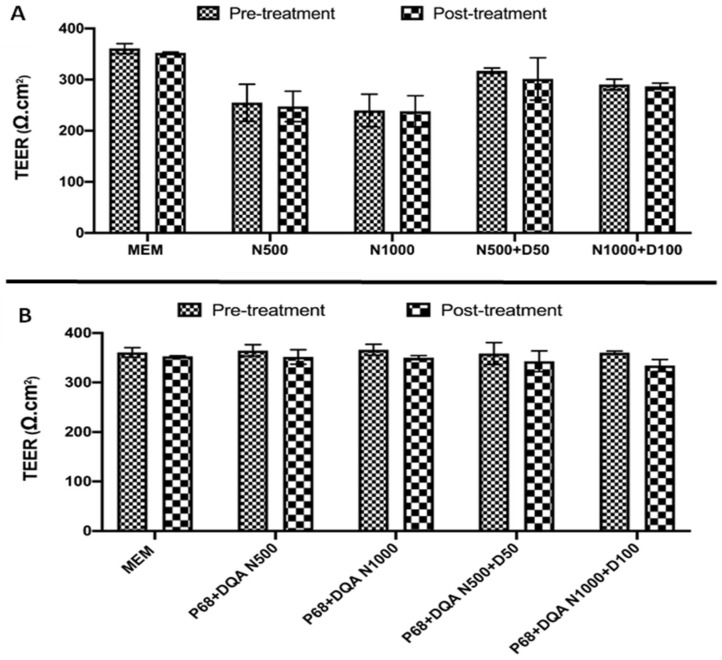
(**A**) Mean TEER of hCMEC/D3 cell monolayers on day 3 post seeding before (pre-treatment) and after (post-treatment) treatment with 500 µM or 1000 µM NAC (N500 and N1000) or the combination of 500 µM or 1000 µM NAC with 50 µM or 100 µM DFO (N500 + D50 and N1000 + D100, respectively). (**B**) Corresponding TEER results for the P68 + DQA nanoformulated NAC and NAC + DFO treatments.

A significant reduction in TEER was observed following treatment with 100 µM free DFO (*p* = 0.0108, Figure 2A). There was no significant change in TEER following treatment with any other free or nanoformulated versions of curcumin or DFO (Figure 2). Similarly, no significant difference in TEER was observed following any of the free and nanoformulated NAC treatments (Figure 3). 

**Figure 4:**
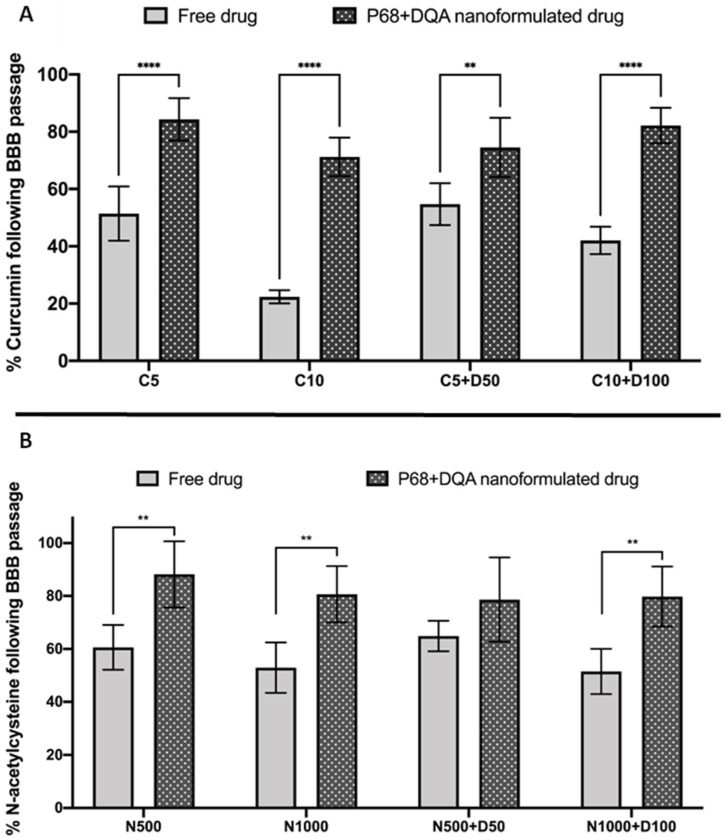
(**A**). Mean percentage of curcumin in the basolateral compartment of the hCMEC/D3 Transwell^®^ system following 60 min treatment with free or P68 + DQA curcumin (5 and 10 µM) and combined curcumin and DFO (5 and 10 µM curcumin + 50 and 100 µM DFO, respectively). (**B**) Mean percentage of NAC in the basolateral compartment of the hCMEC/D3 Transwell^®^ system following 60 min treatment with free or P68 + DQA NAC (500 and 1000 µM) and combined NAC and DFO (500 and 1000 µM NAC + 50 and 100 µM DFO, respectively). Percentage curcumin or NAC = ((absorbance of the basolateral compartment sample − control)/(absorbance of the treatment − control)) × 100, where the absorbance was read at 423 for curcumin or 234 nm for NAC and the control was MEM. * represents significance values of nanoformulated drug compared to free drug within the same treatment condition (**** *p* < 0.0001, ** *p* < 0.01).

When assessing the percentage of curcumin able to pass through the hCMEC/D3 monolayers, significant differences were observed between the different treatment preparations when comparing free and P68 + DQA nanoformulated curcumin (F(1, 32) = 235.4, *p* < 0.0001) (Figure 4A). In the majority of cases, significantly higher percentages of curcumin were reached with the P68 + DQA nanoformulations compared to free curcumin and cur-cumin + DFO treatments (*p* < 0.0001 in all cases, except with P68 + DQA 5 µM curcumin + 50 µM DFO where *p* = 0.0018), with the formulations resulting in between 19.8–48.8% more curcumin compared to the free drug treat-ments (Figure 4A). The largest differences between the formulations and free drug treatments were observed at 10 µM curcumin where the P68 + DQA formulations resulted in 48.8% more curcumin compared to treatment with free curcumin (Figure 4A). However, the highest percentage of curcumin (more than 80%) passing the hCMEC/D3 monolayer was achieved with the 5 µM curcumin and 10 µM curcumin + 100 µM DFO treatments (Figure 4A).

Similarly, when comparing the P68 + DQA nanoformulation and free drug treatments of NAC, and NAC + DFO, significant differences in the percentage of NAC (F(1, 32) = 44.73, *p* < 0.0001) were observed following passage across the hCMEC/D3 monolayer (Figure 4B). The P68 + DQA formulations resulted in significantly higher NAC for all conditions except 500 µM NAC + 50 µM DFO, resulting in between 13.8% and 28.3% more NAC compared to the free drug treatments (500 µM NAC—*p* = 0.0026, 1000 µM NAC—*p* = 0.0024 and 1000 µM NAC + 100 µM DFO—*p* = 0.0079) (Figure 4B). The highest percentage of NAC following passage across the hCMEC/D3 monolayer (88.2%) was achieved using P68 + DQA 500 µM NAC, however all P68 + DQA formulations resulted in more than 78% NAC in the basolateral compartment (Figure 4B). 

**Figure 5:**
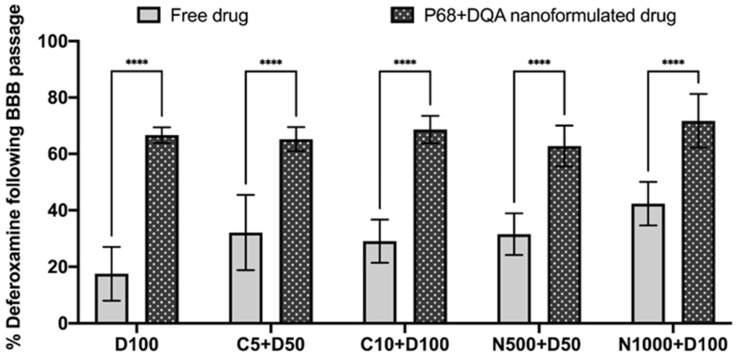
Mean percentage of DFO in the basolateral compartment of the hCMEC/D3 Transwell^®^ system following 60 min treatment with free or P68 + DQA DFO (100 µM), combined curcumin and DFO (5 and 10 µM curcumin + 50 and 100 µM DFO, respectively) and combined NAC and DFO (500 and 1000 µM NAC + 50 and 100 µM DFO, respectively). Per-centage DFO = ((absorbance of the basolateral compartment sample − control)/(absorbance of the treatment − control)) × 100, where the absorbance was read at 204 nm and the control was MEM. * represents significance values of nanoformulated drug compared to free drug within the same treatment condition (**** *p* < 0.0001).

Significant differences in the percentages of DFO passing the BBB monolayer were also observed between the different preparation types, when comparing free and P68 + DQA DFO, curcumin + DFO and NAC + DFO (F(1, 34) = 222.0, *p* < 0.0001) (Figure 5). In all cases, the P68 + DQA nanoformulated preparations resulted in significantly more DFO (between 29.3 and 49.1%) compared to the free drug treatments (*p* < 0.0001 in all cases), with the highest increase of DFO observed when using the P68 + DQA 100 µM DFO treatment (Figure 5).

**Figure 6:**
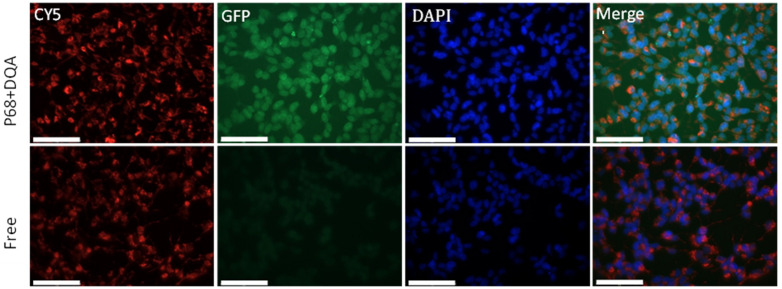
Mitochondrial targeting with P68 + DQA 10 µM curcumin compared to free 10 µM curcumin in SH-SY5Y cells following passage across the hCMEC/D3 Transwell^®^ model. Mitotracker™-stained mitochondria are shown in red (Cy5 objective), internalised curcumin is shown in green (GFP objective); stained nuclei are shown in blue (DAPI objective) and orange indicates the overlap of curcumin and mitochondrial fluorescence (merged image). Scale bars = 75 µm.

Due to the ability of curcumin to autofluoresce, free and P68 + DQA nanoformulated curcumin was used to assess the mitochondrial targeting properties of the P68 + DQA nanocarriers. The fluorescent microscopy imaging presented in Figure 6 shows relatively high levels of curcumin accumulation in SH-SY5Y cells following treatment with P68 + DQA curcumin, indicating high cellular uptake of these nanocarriers following passage across the hCMEC/D3 monolayer. There was minimal curcumin accumulation observed with the free curcumin treatment (Figure 6). The merged image for the P68 + DQA treatment shows significant overlap of curcumin and cell mitochondria fluorescence (Figure 6), indicating co-location of the curcumin released from the P68 + DQA nanocarriers and mitochondria. There was no clear overlap between curcumin and mitochondria with the free curcumin treatment (Figure 6).

**Figure 7:**
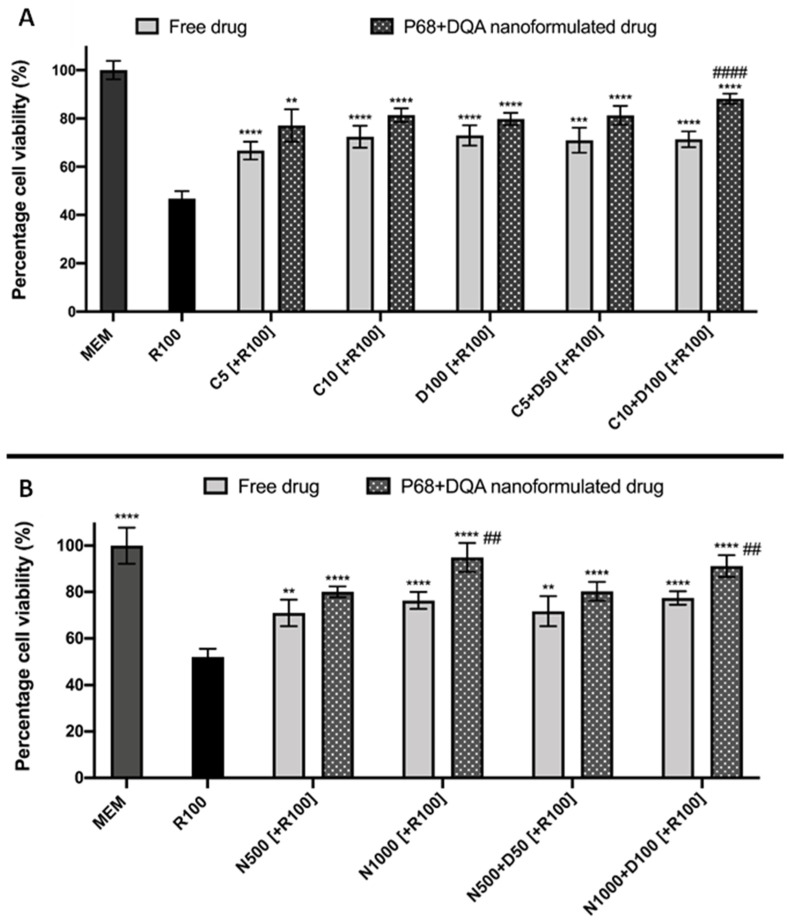
(**A**) SH-SY5Y MTT assay results for free and P68 + DQA preparations of 5 and 10 µM curcumin, 100 µM DFO or combined curcumin and DFO (5 or 10 µM curcumin + 50 or 100 µM DFO, respectively) following passage across the hCMEC/D3—SH-SY5Y co-culture Transwell^®^ system. The hCMEC/D3 cells were grown on the insert and the SH-SY5Y cells were located at the bottom of the basolateral compartment. Treatments were added to the apical compartment of the Transwell^®^ system and incubated for 3 h, the SH-SY5Y cells were then incubated with 100 µM rotenone for 24 h. These results were compared to rotenone treatment alone. MEM represents the control condition where cells were only treated with media (mean ± S.D., n = 6). (**B**) Corresponding MTT assay results for free and P68 + DQA preparations of 500 and 1000 µM NAC and combined NAC and DF0 (500 or 1000 µM NAC + 50 or 100 µM DFO, respectively) following passage across the hCMEC/D3—SH-SY5Y co-culture Transwell^®^ system. * represents significance values of control or pre-treatment conditions compared to rotenone treatment alone (**** *p* < 0.0001, *** *p* < 0.001, ** *p* < 0.01). # represents significance values of nanoformulated drug compared to free drug within the same treatment condition (#### *p* < 0.0001, ## *p* < 0.01).

When assessing the ability of free and P68 + DQA curcumin and curcumin + DFO to protect against rotenone induced cytotoxicity following passage across the hCMEC/D3 monolayer, significant differences were observed (F(11, 42.47) = 95.47, *p* < 0.0001) (Figure 7A). 3 h pre-treatment with all free and formulated curcumin and curcumin + DFO conditions significantly protected against the 53.2% reduction in cell viability induced by rotenone (*p* < 0.0001 in all cases except with free 10 µM curcumin + 100 µM DFO where *p* = 0.0006 and P68 + DQA 5 µM curcumin where *p* = 0.0014). Pre-treatment with P68 + DQA preparations of 10 µM curcumin + 100 µM DFO resulted significantly higher cell viability (16.8%, *p* = 0.0002) compared to the free drug preparation (Figure 7A). The P68 + DQA 10 µM curcumin + 100 µM DFO condition was the most protective against rotenone induced reduction in cell viability, maintaining cell viability at 88.2% of control (Figure 7A). 

Likewise, when comparing free and P68 + DQA NAC treatments, significant differences in cell viability were also observed (F(9, 34.74) = 44.8, *p* < 0.0001) (Figure 7B). All free and P68 + DQA NAC pre-treatments were able to protect against rotenone induced cytotoxicity following passage across the BBB model (*p* < 0.0001 in all cases except with free 500 µM NAC and free 500 µM NAC + 50 µM DFO where *p* = 0.0036 and *p* = 0.005, respectively). However, P68 + DQA 1000 µM NAC and 1000 µM NAC + 100 µM DFO conditions resulted in significantly higher cell viabilities compared to the corresponding free drug conditions (18.6% *p* = 0.0067 and 13.7% *p* = 0.0081, respectively), in both cases maintaining cell viability at more than 90% of control (Figure 7B).

**Figure 8:**
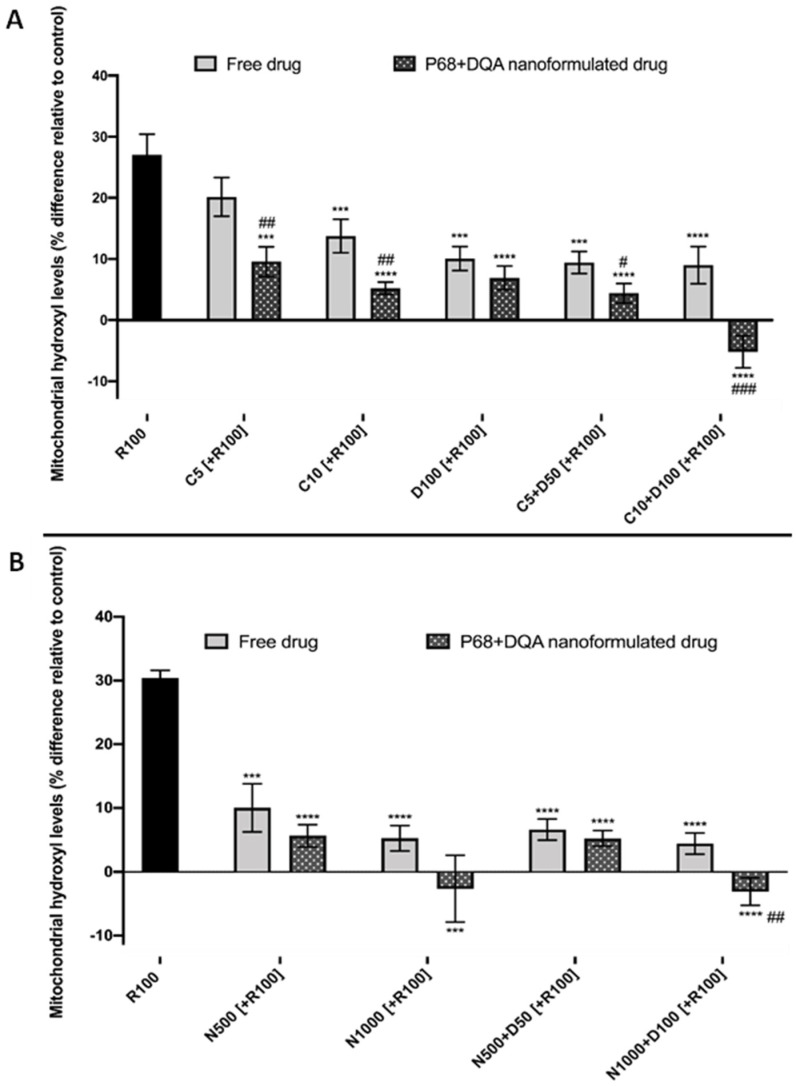
(**A**) SH-SY5Y mitochondrial hydroxyl radical assay results for free and P68 + DQA preparations of 5 and 10 µM curcumin, 100 µM DFO or combined curcumin and DFO (5 or 10 µM curcumin + 50 or 100 µM DFO, respectively) following passage across the hCMEC/D3—SH-SY5Y co-culture Transwell^®^ system. The hCMEC/D3 cells were grown on the insert and the SH-SY5Y cells were located at the bottom of the basolateral compartment. Treatments were added to the apical compartment of the Transwell^®^ system and incubated for 3 h, the SH-SY5Y cells were then incubated with 100 µM rotenone for 24 h. These results were compared to rotenone treatment alone. Mitochondrial hydroxyl radical levels are expressed as the percentage of hydroxyl radical identified in control cells (SH-SY5Y cells treated with MEM media only, for 24 h). (mean ± S.D., n = 6). (**B**) Corresponding mitochondrial hydroxyl radical assay results for free and P68 + DQA preparations of 500 and 1000 µM NAC and combined NAC and DF0 (500 or 1000 µM NAC + 50 or 100 µM DFO, respectively). * represents significance values of control or pre-treatment conditions compared to rotenone treatment alone (**** *p* < 0.0001, *** *p* < 0.001). # represents significance values of nanoformulated drug compared to free drug within the same treatment condition (### *p* < 0.001, ## *p* < 0.01, # *p* < 0.05).

Mitochondrial hydroxyl radical levels were also assessed using the Transwell^®^ model to evaluate the ability of the free and nanoformulated treatments to protect against rotenone induced oxidative stress. Significant differences were observed between the different free and P68 + DQA curcumin and curcumin + DFO treatments (F(11, 42.75) = 71.23, *p* < 0.0001) (Figure 8A). All curcumin and curcumin + DFO free and P68 + DQA conditions, except 5 µM free curcumin, significantly protected against rotenone induced increased hydroxyl radical levels (free drug: 10 µM curcumin—*p* = 0.0008, 100 µM DFO—*p* = 0.0002, 5 µM curcumin + 50 µM DFO—*p* = 0.0001 and 10 µM curcumin + 100 µM DFO—*p* < 0.0001. P68 + DQA: *p* ≤ 0.0001 in all cases) (Figure 8A). 

In each case, the P68 + DQA preparations were the most protective, resulting in lower percentage hydroxyl radical levels in all cases compared to the corresponding free drug conditions (between 3.2 and 14.2%) (Figure 8A), with P68 + DQA 10 µM curcumin + 100 µM DFO resulting in the lowest hydroxyl radical levels, −5.2% relative to control (Figure 8A). The differences in hydroxyl radical were found to be significantly lower when pre-treating with P68 + DQA preparations of 5 µM curcumin (*p* = 0.004), 10 µM curcumin (*p* = 0.0091), 5 µM curcumin + 50 µM DFO (*p* = 0.0169) and 10 µM curcumin + 100 µM DFO (*p* = 0.0003) compared to the corresponding free drug conditions (Figure 8A). 

Significant differences were also observed when using the mitochondrial hydroxyl radical assay to assess the ability of free and P68 + DQA NAC and NAC + DFO (F(8, 18.79) = 83.49, *p* < 0.0001) to protect against rotenone induced oxidative stress in the Transwell^®^ model (Figure 8B). All free and P68 + DQA NAC and NAC + DFO conditions significantly protected against the rise in hydroxyl radical induced by rotenone (*p* ≤ 0.0001 in all cases, Figure 8B). Again, the P68 + DQA conditions generally resulted in lower hydroxyl radical compared to the free drug conditions (Figure 8B). However, of the NAC and NAC + DFO treatments, the only significant difference between the P68 + DQA and free drug preparations was with 1000 µM NAC + 100 µM DFO (*p* = 0.0022), where there was a difference of 7.5%, with the percentage of hydroxyl radical following the P68 + DQA treatment reaching −3.1% relative to control levels (Figure 8B).

## 4. Discussion

The human derived brain endothelial cell line, hCMEC/D3, cultured on permeable cell culture plate inserts is a well-established *in vitro* model of the BBB [50,51,61,62]. This model can be used in co-culture with other cell lines to evaluate treatments following permeation of the membrane to provide a more accurate indicator of the potential *in vivo* effects [62,63]. This study combined the hCMEC/D3 BBB model with the SH-SY5Y rotenone model of PD in a Transwell^®^ co-culture system to evaluate the passage of P68 + DQA nanoformulated curcumin, NAC, and/or DFO treatments across the model BBB as well as the effectiveness of these treatments at protecting against PD-related neurotoxicity and oxidative stress following permeation of the membrane. For a preclinical screening study of this nature, the model has the advantage of allowing quantitative permeability assessments in combination with real-time live cell determinations while limiting use of animals. Although not a replacement for animal models, this approach allows selection and refinements of the most optimal delivery systems which can then be fully validated in *in vivo* studies.

The TEER of the hCMEC/D3 monolayers peaked at 320 Ω.cm^2^. This, along with the mean permeability of 0.91 ± 0.13 × 10^−3^ cm/min, was consistent with previous studies which have reported acceptable permeability as less than 1.2 × 10^−3^ cm/min [55,56,57] and TEER values of approximately 300 Ω.cm^2^ when cultured in the presence of hydrocorti-sone to modulate the expression of tight junctional proteins and prevent endothelial barrier breakdown [48,51,52,56]. 

The various free and nanoformulated curcumin and NAC treatments, alone and in combination with DFO, were tested on this model to assess whether they were likely to enter the brain *in vivo* and to evaluate whether the protective effects of these treatments against rotenone induced oxidative stress (as previously outlined by Mursaleen et al. [38,39]) were retained after passage across a model of the BBB. Importantly, no significant differences in TEER values were observed following treatment with all free and P68 + DQA curcumin, NAC and combined DFO conditions, suggesting that none of these treatments are likely to cause toxicity to the BBB (Figure 2 and Figure 3). 100 µM free DFO did however cause a significant reduction in TEER and therefore may not be suitable as a treatment for PD. The fact that 100 µM free DFO resulted in some cytotoxicity of the hCMEC/D3 cells but the corresponding nanoformulated treatments did not, suggests that the formulation strategy is protective against this effect. Furthermore, none of the combination treatments containing 100 µM DFO resulted in any cytotoxicity, including the free drug conditions, suggesting that in this case the presence of the antioxidant in the free drug treatments may act as a protective agent to counter the detrimental effects of the high DFO concentration. However, if this were to be the case in a physiological scenario, a proportion of the total overall antioxidant power of the free drug conditions may be consumed at the site of the BBB, reducing the activity available at the desired site of neurodegeneration. This further highlights the benefit of nanoformulation for these drugs to enable targeted delivery.

In line with previous literature [22,63,64,65,66], these results indicate that curcumin, DFO and NAC can all pass the BBB to some extent. However, in every case the P68 + DQA nanoformulation of these drugs increased the percentage of each antioxidant reaching the basolateral compartment of the Transwell^®^ model by up to 49% for curcumin (with 10 µM curcumin) and 28% for NAC (with 1000 µM NAC and 1000 µM NAC + 100 µM DFO) (Figure 4). This trend was also observed for treatments containing DFO, where the percentage of DFO passing the BBB model reached up to 72% with the combined P68 + DQA NAC + DFO (Figure 5). 

Although it was previously reported that DQA is a mitochondrial targeting agent [32,40,41]; the live cell mitochondrial staining analysis results presented here have demonstrated for the first time that the P68 + DQA nanoformulations resulted in more curcumin released at the mitochondria compared to free curcumin treatment (Figure 6). This indicates mitochondrial-specific accumulation of curcumin when using the P68 + DQA nanocarriers due to the ability of DQA to specifically target cellular mitochondria. The P68 + DQA nanocarriers may therefore be particularly suitable for PD because mitochondria are the main site of oxidative stress [12,14,15,42] and mitochondrial dysfunction has been linked to the development and progression of PD [67,68,69,70,71,72,73,74]. This is further supported by the SH-SY5Y cell viability and mitochondrial hydroxyl radical assay results which show that the P68 + DQA formulations of each treatment consistently resulted in the most protection against rotenone, which is a mitochondrial complex 1 inhibitor [75], following passage across the hCMEC/D3 Transwell^®^ model. 

Overall, the cell viability and mitochondrial hydroxyl radical assay results were consistent with previous studies which have shown protective effects of curcumin, NAC and DFO against rotenone in *in vitro* models of PD [18,21,38,39]. However, unlike the results reported by Mursaleen et al. [38] where the P68 + DQA curcumin nanoformulations were assessed in SH-SY5Y cells alone without the BBB Transwell^®^ compartment and where for the most part the formulations were only at least as good as the free drug conditions at protecting against rotenone, when using the Transwell^®^ model the P68 + DQA nanoformulated treatments were found to be generally more protective than the free drugs. In line with the live cell mitochondrial staining analysis, this suggests that the P68 + DQA formulations were able to mostly stay intact until reaching the mitochondria within the SH-SY5Y cells where they could then exert their effects, highlighting the value of the targeted delivery approach. 

There was, however, little change compared to previous reports using the SH-SY5Y model alone in terms of which concentrations of these treatments were the most successful at protecting against rotenone [38,39]. The highest concentrations of the P68 + DQA combinations of curcumin and DFO were the most effective of the treatments containing curcumin at protecting against rotenone induce cytotoxicity and increased mitochondrial hydroxyl, maintaining cell viability above 80% and hydroxyl radical at least in line with control levels (Figure 7A and Figure 8A). Of the treatments containing NAC, P68 + DQA 1000 µM NAC was equally as effective as the combination of 1000 µM NAC with 100 µM DFO, in each case maintaining cell viability above 91% and hydroxyl radical 2.7% below control levels (Figure 7B and Figure 8B). As implied by previous work [38,39], these results further suggest that NAC, unlike curcumin, does not necessarily benefit from the addition of an iron chelator, likely due to its potent iron chelating capabilities [76,77,78]. Although curcumin has also been reported to possess metal chelating properties forming ligand:metal complexes in a 2:1 ratio [79], these effects may not be realised at the low treatment concentrations used for curcumin compared to those of NAC. This may be because at such high metal iron concentrations the α,β-unsaturated β-diketo moiety of curcumin that is known to be responsible for forming metal chelates may have been fully utilised [79]. Thus, at these curcumin concentrations the inclusion of a chelator such as DFO could be expected to have been beneficial.

## 5. Conclusions

In summary, taken together, these results indicate that the P68 + DQA nanoformulations were successful at enhancing the protective effects of curcumin and NAC, alone and in combination with DFO, by increasing the BBB permeability and targeted delivery of the associated drugs within the cellular Transwell^®^ system. This is supported by the live cell mitochondrial staining analysis results which demonstrated for the first time mitochondrial-specific accumulation of curcumin when using the P68 + DQA nanocarriers in a Transwell^®^ hCMEC/D3—SH-SY5Y co-culture system. The improved biological effects of protecting against rotenone observed with the P68 + DQA nanoformulations compared to the free drug treatments in this system is supportive of these nanocarriers increasing passage across the model BBB. This nanocarrier strategy may therefore be an effective approach to fully utilise the therapeutic benefit of these antioxidants. The use of lipid nanoparticles to deliver the Pfizer-BioNTech [80] and the SARS-CoV-2 [81] COVID-19 vaccinations recently has made great progress in promoting the use of nanocarrier delivery systems in clinical therapy. Such work paves the way for nanocarriers, such as the P68 + DQA formulations evaluated in this study, to be potentially used for clinical therapy, after comprehensive evaluations have first been made in more advanced models of disease.

## Figures and Tables

**Table 1:** Hydrodynamic Diameter (d), Polydispersity Index (PDI), Surface Charge, Drug Loading (DL) and Association Efficiency (AE) of blank and drug-loaded P68 + DQA nanoformulations prepared at and 80 °C (mean ± S.D., n = 6) as previously reported in Mursaleen et al. [34,35].

Sample	Contents (mg/mL)		d (nm)	PDI	Charge (mV)	DL (%)	AE (%)
P68 + DQA (Blank)	P68:	9	25.52 ± 10.25	0.24 ± 0.04	−0.78 ± 0.80	-	-
	DQA:	1					
P68 + DQA	P68:	9	182.6 ± 31.5	0.10 ± 0.09	4.27 ± 4.15	14.68 ± 1.55	86.17 ± 10.58
Curcumin	DQA:	1					
	Curcumin:	2					
P68 + DQA	P68:	9	191.8 ± 45.3	0.08 ± 0.04	9.29 ± 5.12	Curcumin:	Curcumin:
Curcumin + DFO	DQA:	1				7.36 ± 0.19	81.78 ± 10.16
	Curcumin:	0.28				DFO:	DFO:
	DFO	5				31.77 ± 1.80	95.56 ± 7.83
P68 + DQA	P68:	9	50.44 ± 33.1	0.25 ± 0.05	0.02 ± 1.62	16.10 ± 0.43	95.94 ± 3.07
DFO	DQA:	1					
	DFO:	2					
P68 + DQA	P68:	9	125.67 ± 9.98	0.23 ± 0.05	3.67 ± 0.46	64.88 ± 1.93	92.74 ± 7.54
NAC	DQA:	1					
	NAC:	20					
P68 + DQA	P68:	9	130.33 ± 11.49	0.24 ± 0.02	6.63 ± 1.44	NAC:	NAC:
NAC + DFO	DQA:	1				17.53 ± 0.56	98.32 ± 1.44
	NAC:	12.4				DFO:	DFO:
	DFO:	5				17.59 ± 0.54	94.36 ± 4.27

## Data Availability

The data presented in this study are available on request from the corresponding author.
